# *Paracapillaria* (*Ophidiocapillaria*) *siamensis* sp. nov. (Nematoda: Trichuroidea): a new nematode in *Naja kaouthia* from Thailand

**DOI:** 10.1017/S0031182024000404

**Published:** 2024-04

**Authors:** Vachirapong Charoennitiwat, Kittipong Chaisiri, Tapanee Kanjanapruthipong, Sumate Ampawong, Lawan Chanhome, Taksa Vasaruchapong, Urusa Thaenkham, Napat Ratnarathorn

**Affiliations:** 1 Department of Helminthology, Faculty of Tropical Medicine, Mahidol University, Bangkok, Thailand; 2 Department of Tropical Pathology, Faculty of Tropical Medicine, Mahidol University, Bangkok, Thailand; 3 Snake Farm, Queen Saovabha Memorial Institute, The Thai Red Cross Society, Bangkok, Thailand; 4 Animal Systematics & Molecular Ecology Laboratory and Applied Animal Science Laboratory, Department of Biology, Faculty of Science, Mahidol University, Bangkok, Thailand

**Keywords:** gastrointestinal helminth, monocled cobra, morphological and molecular analysis, *Naja kaouthia*, *Ophidiocapillaria*, *Paracapillaria siamensis*

## Abstract

A comprehensive investigation, incorporating both morphological and molecular analyses, has unveiled the existence of a hitherto unknown nematode species, *Paracapillaria* (*Ophidiocapillaria*) *siamensis* sp. nov., residing in the intestine of the monocled cobra, *Naja kaouthia*, in the central region of Thailand. This study integrates morphological characteristics, morphometric examination, scanning electron microscopy and molecular phylogenetic analysis (*COI*, 18S rRNA and ITS1 genes). The findings place the newly described species within the subgenus *Ophidiocapillaria*, elucidating its distinctive characteristics, including a frame-like proximal spicule shape, approximate lengths of 19 000 and 22 500 μm with approximate widths of 90 and 130 μm for males and females, 39‒45 stichocytes, elevated lips without protrusion, a dorsal bacillary band stripe with an irregular pattern of bacillary cells and evidence of intestinal infection. These features serve to differentiate it from other species within the same subgenus, notably *Paracapillaria* (*Ophidiocapillaria*) *najae* De, 1998, a species coexisting *P*. *siamensis* sp. nov. in the monocled cobra from the same locality. This study addresses the co-infection of the novel species and *P. najae* within the same snake host, marking the second documented instance of a paracapillariid species in the monocled cobra within the family Elapidae. The genetic characterization supports the formal recognition of *P*. *siamensis* sp. nov. as a distinct species, thereby underscoring its taxonomic differentiation within the Capillariidae family. This research identifies and characterizes the new nematode species, contributing valuable insights into the taxonomy of this nematode.

## Graphical abstract

**Figure:**
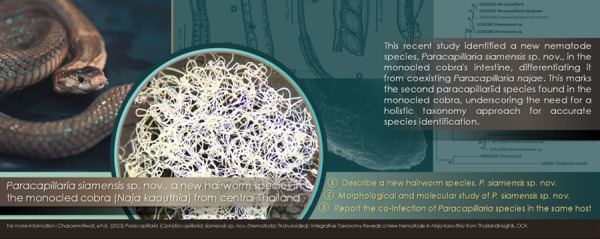

## Introduction

The nematode genus *Paracapillaria* Mendonça, 1963, which belongs to the family Capillariidae Railliet, 1915, has been classified into 3 distinct subgenera: *Paracapillaria* Mendonça 1963, found in fishes and amphibians; Ophidiocapillaria Moravec, 2001, which inhabits reptiles and *Crossicapillaria* Moravec, 2001, known to infect avian and mammalian hosts (Moravec, 1986; Moravec and Justine, 2020). One particular species within *Crossicapillaria*, *Paracapillaria* (*Crossicapillaria*) *philippinensis* (Chitwood, Velázquez y Salazar, 1968), has garnered the attention of the Centers for Disease Control and Prevention (CDC) due to its potential for zoonotic transmission, signifying its capacity to induce intestinal capillariasis in humans (Chitwood *et al*., 1968; El-Karaksy *et al*., 2004; Saichua *et al*., 2008). While substantial research has been conducted on the medically important worms, the subgenus *Ophidiocapillaria*, particularly within snakes, remains relatively underexplored from all angles, including its basic information like classification (Moravec, 1986; Moravec and Justine, 2020).

Until recently, researchers have identified 15 distinct species within *Ophidiocapillaria* inhabiting various snake host species. These encompass *Paracapillaria mimgazzinii*, described in the grass snake, *Natrix natrix* (Skryabin *et al*., 1957); *Paracapillaria xochimilcensis*, described in the narrow-headed garter snake, *Thamnophis rufipunctatus* (Skryabin *et al*., 1957); *Paracapillaria sonsinoi*, described in the green whip snake, *Hierophis viridiflavus* (Skryabin *et al*., 1957), alongside the viperine water snake, *Natrix maura*, and the diamondback water snake, *Nerodia rhombifera* (Moravec, 1986); *Paracapillaria heterodontis*, described in the eastern hog-nosed snake, *Heterodon platirhinos* (Pence, 1970); *Paracapillaria colubra*, described in the eastern racer, *Coluber constrictor* (Pence, 1970); *Paracapillaria viperae*, described in the nose-horned viper, *Vipera ammodytes* (Biserkov *et al*., 1985); *Paracapillaria ptyasi*, described in the oriental rat snake, *Ptyas mucosa* (Wang, 1982); and *Paracapillaria najae*, described in the Indian cobra, *Naja naja* (De, 1998) and the monocled cobra, *Naja koauthia* (Charoennitiwat *et al*., 2023). Throughout most of these investigations, the primary emphasis was on morphological criteria for species identification, with microscopic structure and molecular data receiving comparatively limited attention.

Between 2000 and 2023, authors of this research conducted parasitological surveys in wildlife (Unpublished data), leading to the discovery of 2 distinct paracapillariid species residing within various digestive organs of the monocled cobras, *Naja kaouthia*, in central regions of Thailand. Additionally, other studies have reported finding eggs from these worms within the aforementioned snake host and location. However, precise identification has posed a considerable challenge, resulting in a taxonomic classification limited to the genus level, denoted as *Capillaria* sp. (Chaiyabutr and Chanhome, 2002; Vasaruchapong *et al*., 2017). The morphological similarity between these 2 paracapillariid species, without genetic investigation, caused confusion and led to the erroneous assumption in the survey that they were the same species. One of these species, *P. najae*, located in the cobra's oesophagus, was initially described by De in 1998 and subsequently redescribed by Charoennitiwat *et al*. (2023). Conversely, the other species, infesting the cobra's intestine, exhibited discernible variations in both morphology and genetic sequences when compared to the data documented for *P. najae*, as delineated by Charoennitiwat *et al*. (2023). This comprehensive examination ultimately resulted in the formal recognition of these as distinct new species and the revelation of the co-infection of this novel species alongside *P. najae* in the same snake host species.

This led to the inception of the present study. A substantial number of *Paracapillaria* samples retrieved from the monocled cobra's intestine underwent detailed molecular phylogenetic analysis and thorough morphological examination. The scrutiny of external morphological characteristics aimed to distinguish the new nematode species within its taxonomic context, aligning with insights derived from genetic data. The exploration of phylogenetic relationships involved the reconstruction utilizing genes, including cytochrome c oxidase subunit 1 (*COI*), internal transcribed spacer1 (ITS1) and small subunit nuclear ribosomal RNA (18S rRNA). Our findings conclusively validate the presence of the newly described *Paracapillaria* (*Ophidiocapillaria*) *siamensis* sp. nov., reinforcing previous reports of co-infection incidents involving *P. najae* in the same hosts organisms.

## Materials and methods

### Specimen preparation

Five carcasses of wild monocled cobras were provided by the Snake Farm at Queen Saovabha Memorial Institute, Thai Red Cross Society in Bangkok, Thailand. These cobras underwent dissection to investigate the presence of parasites in their intestine, following the protocols outlined by Toland and Dehne (1960) as well as Terrell and Stacy (2007). Following the dissection, the organs were placed in Petri dishes filled with 0.85% normal saline and were subsequently examined under stereo-microscopes (Olympus, SZ30 and SZ51, Japan). Micro-dissecting needles were employed to observe and isolate the parasites, focusing on the paracapillariid nematodes. These isolated parasites were counted and preserved in 70% ethanol at −20 °C until required. Additionally, 4 specimens were separated and preserved in glutaraldehyde for scanning electron microscopic imaging.

### Morphological study

For the morphological studies, 21 male and 21 female intact worm specimens were selected from the preserved 70% ethanol stock and subjected to comprehensive examination. This examination was conducted using an inverted microscope (Zeiss, Primovert, Germany) equipped with a Zeiss Axiocam and ZEN2 blue edition software. The measurements in micrometers (μm) of these morphological characters strictly adhered to the methodologies outlined by Biserkov *et al*. (1994), De (1998) and Charoennitiwat *et al*. (2023). Illustrations depicting selected morphological traits of both male and female worms were crafted under a light microscope with a camera lucida (Leitz, Wetzlar, Germany). Out of the total number of specimens, after the morphological investigations, 26 specimens (13 males and 13 females) were initially mounted in glycerine jelly and subsequently double mounted again with permount to establish permanent slides of type specimens. The remainder of the specimens (8 specimens for each sex) were designated for genetic analysis.

### Scanning electron microscopy study

For a scanning electron microscope (SEM) analysis, 2 intact male and 2 intact female specimens were carefully selected. Initially, these specimens were immersed in a solution containing 2.5% glutaraldehyde in a 0.1 M sucrose phosphate buffer (SPB) for primary fixation. Subsequently, a secondary fixation step was performed using a 1% osmium tetroxide solution in the same 0.1 M SPB. The specimens were then dehydrated with ethanol and subsequently dried using a critical point drying device (CPD300 auto, Leica, Wetzlar, Germany). A fine coating of material was applied using a coat-sputter (Q150R PLUS, Quorum, East Sussex, England). Finally, these prepared specimens were examined under the SEM (JSM-6610LV, JEOL, Tokyo, Japan). This SEM analysis was conducted at the Department of Tropical Pathology, Faculty of Tropical Medicine, Mahidol University.

The taxonomic classification of the specimen was conducted based on the morphology outlined by Moravec and Justine (2020). Morphological comparisons among paracapillariid species in the subgenus *Ophidiocapillaria* were made using information from various sources, including Teixeira de Freitas and Lent (1934), Skryabin *et al*. (1957), Pence (1970), Wang (1982), Biserkov *et al*. (1985), Moravec and Gibson (1986), Moravec (1986), De (1998) and Charoennitiwat *et al*. (2023), with reference examined specimens listed in Table S1.

### Molecular study

For DNA extraction, 8 male and 8 female specimens were homogenized and processed using the DNeasy Blood & Tissue Kit (Qiagen, Germany) following the manufacturer's instructions. The extracted genomic DNA was eluted with 30 μL of nuclease-free water and quantified using spectrophotometry.

A partial mitochondrial (mtDNA) *COI* gene sequence was amplified from the selected samples. This gene locus has been previously demonstrated to be valuable for elucidating genetic diversity within nematode species (Chan *et al*., 2020). Additionally, nuclear genes, 18S rRNA and ITS1, were amplified to support the results of the mtDNA analysis (Tokiwa *et al*., 2012; Eamsobhana *et al*., 2015; Chan *et al*., 2020). The following primers were used: COI_Paracap_F 5′-AGTRTTTGGTCCTTTRGG-3′ and COI_Paracap_R 5′-GAWGCATTAG AAAGAGA-3′ for the *COI* gene, 1096F 5′-GGTAATTCTGGAGCTAA TAC-3′ and 1916R 5′-TTTACGGTCAGAACTA GGG-3′ for the 18S rRNA gene and ITS1_Paracap_F 5′-TGCA GTGCCACCGTCACATC-3′ and ITS1_Paracap_R 5′-GTCAACCGACACGGATTAGC-3′ for the ITS1 gene. The resulting *COI*, 18S rRNA and ITS1 amplicon lengths were 288 bp, 800 bp and 521 bp, respectively.

The PCR reactions were conducted using a T100 thermocycler (Bio-Rad, California, the United States of America). The PCR reaction mixture contained a final volume of 30 μL, including 15 μL of 2X i-Taq master mix (Biotechnology, Gyeonggi, South Korea), 10 μM of each primer and 1 ng/μL of DNA. For the *COI* primers, the thermocycling profile was as follows: an initial denaturation step at 95 °C for 5 min, followed by 30 cycles of 95 °C for 30 s, 52 °C for 1 min and 72 °C for 45 s. The reaction concluded with a final extension step at 72°C for 5 min, as described by Charoennitiwat *et al*. (2023). For the ITS1 primers, the thermocycling profile was as follows: an initial denaturation step at 95 °C for 5 min, followed by 30 cycles of 94 °C for 40 s, 60 °C for 1 min and 72 °C for 2 min. The reaction concluded with a final extension step at 72 °C for 5 min. Regarding the 18S rRNA primers, the thermocycling profile consisted of an initial denaturation at 94 °C for 5 min, followed by 5 cycles of 94 °C for 30 s, 45 °C for 30 s and 72 °C for 70 s, and 35 cycles of 94 °C for 30 s, 54 °C for 30 s and 72 °C for 70 s, as described by Holterman *et al*. (2006).

For the visualization of the PCR amplicons, a 1% agarose gel stained with SYBR safe (Thermo Fisher Scientific, Waltham, the United States of America) was employed. Following this, the PCR products from the 3 most exemplary sample sequences underwent Barcode Taq sequencing, a method that does not necessitate primer walking (Celemics, Seoul, South Korea). The nucleotide sequences derived from the parasite specimens in this study have been submitted to the NCBI database and allocated the respective accession numbers: OR839870–72 for the *COI*, OR840538–40 for the 18S rRNA and OR840543–45 for the ITS1.

The partial sequences of the 3 target genes were verified through manual inspection of electropherograms. The complementary strands were compared and adjusted using BioEdit version 7.2.5 (Hall, 1999). For the analysis of phylogenetic relationships, a phylogenetic tree was constructed using the obtained sequences and additional sequences from parasites of Capillariidae, Trichuridae and/or Trichinellidae retrieved as outgroups from GenBank. The alignment of the data matrix was performed using ClustalX 2.1 (Hall, 1999; Thompson *et al*., 2002), and the aligned sequences were visually analysed using BioEdit. The aligned sequences were verified before conducting the phylogenetic analysis using maximum likelihood (ML) in MEGA-X (Chan *et al*., 2020). The best-fit nucleotide substitution model was determined by the Bayesian Information Criterion (BIC), and the analysis was performed with 1000 bootstrap replicates (Tamura *et al*., 2013). A bootstrap value exceeding 70% generally provides strong support (Hillis and Bull, 1993). The phylogenetic trees were constructed using the ML method, employing the Hasegawa-Kishino-Yano (HKY) model for the ITS1, Kimura 2-parameter (K2) with a gamma distribution for the 18S rRNA and the General Time Reversible (GTR) model with a gamma distribution for the *COI*.

## Results

### Taxonomy

#### Phylum: Nematoda Diesing, 1861 Class: Enoplea Inglis, 1983 Order: Trichocephalida Hall, 1916 Family: Capillariidae Railliet, 1915 Genus: *Paracapillaria* Mendonça, 1963 Subgenus: *Ophidiocapillaria* Moravec, 2001 Species: *Paracapillaria* (*Ophidiocapillaria*) *siamensis* sp. nov. Charoennitiwat *et al*., 2023 (Table 1, Figs 1 and 2) **Type-host**: *Naja kaouthia* Lesson, 1831. **Type-locality**: Bangkok's suburbs (e.g. Don Mueang district, Prawet district, *etc*.) in Thailand. Coordinates were not recorded. **Collection date**: November 10, 2020, to July 18, 2023. **Site of infection**: Small intestine **Parasite intensity**: 16–82 (on average 47) nematodes in the five-host examined **ZooBank LSID:** 7072F98E-09EC-4D3C-BC10-B86D654A7C60 **Etymology**: The specific epithet ‘*siamensis*’ indicates that the nematode species is found in Thailand or belongs to Thailand. ‘*Siam*’ is an original and informal name for ‘Thailand,’ and the suffix ‘-*ensis*’ in Latin is commonly used to create adjectives that signify ‘*of*’ or ‘*belonging to*’ specific places or localities.

### General description

**Figure 1.**
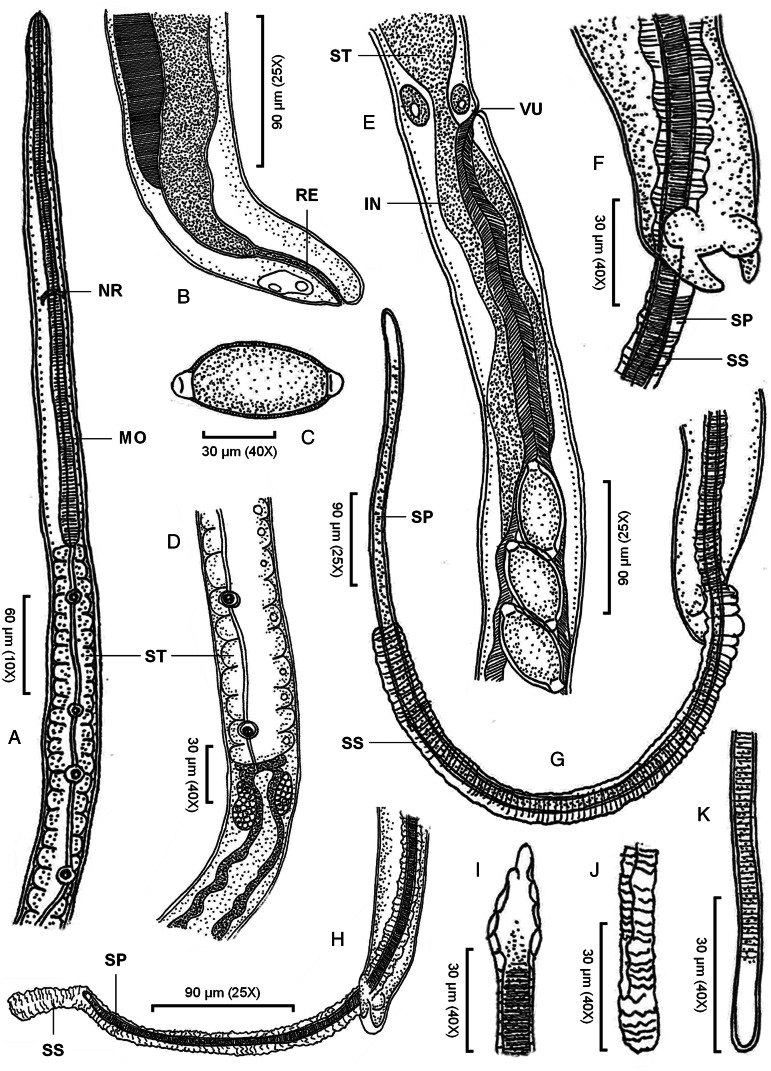
*Paracapillaria* (*Ophidiocapillaria*) *siamensis* sp. nov.: (A) anterior end of male, lateral view; (B) posterior end of female, lateral view; (C) fully developed egg; (D) stichosome region of male; (E) oesophago-intestinal junction of female, lateral view; (F) posterior end of male, enlarged latero-ventral view; (G) posterior end of male, latero-ventral view; (H) posterior end of male, lateral view; (I) proximal part of spicule; (J) spicular sheath; and (K) distal part of spicule. (IN, intestine; MO, muscular oesophagus; NR, nerve ring; RE, rectum; SP, spicule; SS, spicular sheath; ST, stichosome; VU, vulva)

**Figure 2.**
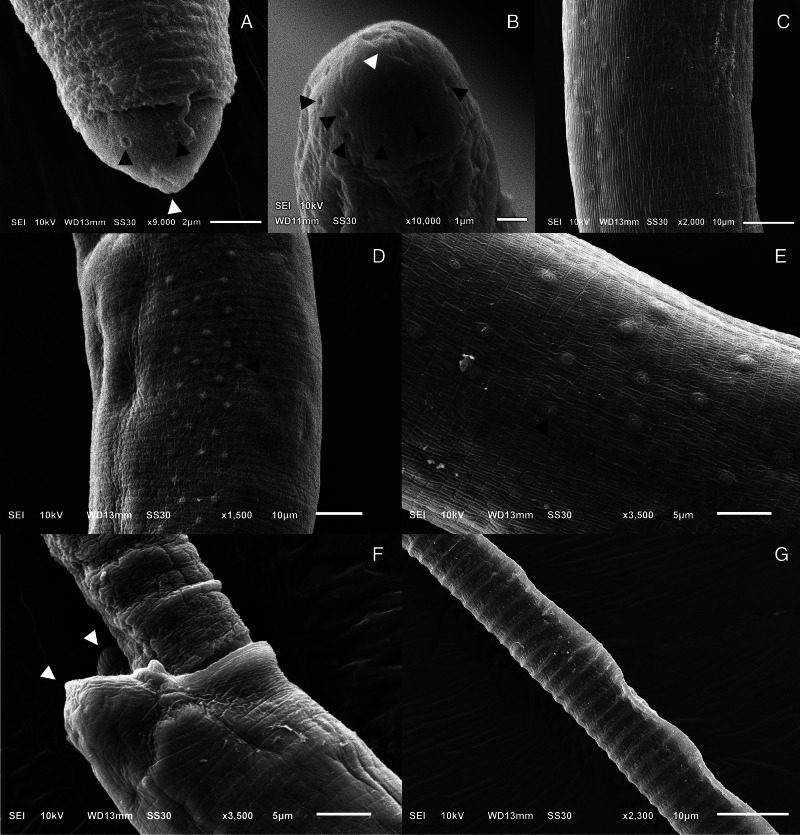
Scanning electron micrograph of *Paracapillaria* (*Ophidiocapillaria*) *siamensis* sp. nov.: (A) lateral view and (B) apical view of head region of male, elevated oral lip (white arrow) surrounded by papillae (black arrows); (C) lateral view of middle body with transverse striations; (D) dorsal view of middle body with a bacillary band and (E) button-like bacillary cells (black arrow); (F) lateral view of posterior end of male with two post-cloacal papillae (white arrows); (G) spicular sheath without spines.

Small-to-medium sized, thin and filiform nematodes. Elevated oral opening surrounded by distinct papillae (Figs 2A‒B). Head narrow and rounded (Figs 1A, 2A‒B). Body long and slender with smooth transversely striated cuticles (Figs 2C‒E). Males shorter and thinner than gravid females. A bacillary band with irregular arrangement of button-like bacillary cells present dorsally, extending between 1/10 of body length and cloaca (Figs 2D‒E). Oesophagus with sinuous muscular part followed by stichosome; stichosome consisting of single row of 39‒45 stichocytes; anterior stichocytes short; middle and posterior stichocytes long and subannulated with large nuclei (Figs 1A, 1D). Stichocytes contain prominent nucleolus with distinct corpuscles (Fig. 1D). Nerve ring encircling muscular oesophagus 2/10 of its length. Two large cells present at oesophago-intestinal junction (Fig. 1E).

**Table 1.** The comparison between *Paracapillaria* (*Ophidiocapillaria*) *siamensis* sp. nov. and *Paracapillaria* (*Ophidiocapillaria*) *najae*

	Charoennitiwat *et al*. (2023): *Paracapillaria* (*O.*) *najae*		Present study: *Paracapillaria* (*O.*) *siamensis* sp. nov.	
Host species	*Naja kaouthia*		*Naja kaouthia*	
Locality	Thailand		Thailand	
Organ	Oesophagus		Intestine	
No. of bacillary band—position	1—dorsolaterally		1—dorsal	
Oral lips	Elevated with protrusion		Elevated without protrusion	
Male	*n* = 21		*n* = 21	
	Ranges	Average ± s.d.	Ranges	Average ± s.d.
Body length	11 579– 25 592	19 069 ± 3902	7150–18 098	13 673 ± 3242
Maximum width	56–123	93 ± 17	53–86	65 ± 8
Nerve-ring from anterior end	44–62	51 ± 6	23–69	50 ± 13
Muscular oesophagus	210–374	287 ± 50	160–395	275 ± 91
Stichosome	4635–9016	6630 ± 1091	3570–9081	6915.27 ± 1363
Entire oesophagus (anterior part)	4855–9344	6916 ± 1116	3805–9452	719 ± 1376
Length of posterior part	6724–18 085	12 381 ± 3158	1663–10 645	6480 ± 2309
Ratio posterior: anterior ends	1.2–2.5	1.8 ± 0.4	0.3–2.0	0.9 ± 0.4
Tail	14–38	24 ± 6	12–23	17 ± 3
Spicule length	1437–2715	2032 ± 327	749–1815	1381 ± 298
Ratio whole body: spicule length	7.9–11.4	9.4 ± 1.1	6.3–20.8	10.1 ± 2.9
Spicule form	Spatula-like		Frame-like	
Female	*n* = 20		*n* = 21	
	Ranges	Average ± s.d.	Ranges	Average ± s.d.
Body length	16 532–31 485	22 518 ± 4465	11 816–25 160	19 630 ± 3862
Maximum width	101–180	133 ± 21	50–112	87 ± 15
Nerve-ring from anterior end	42–66	50 ± 8	40–69	51 ± 9
Muscular oesophagus	148–436	305 ± 71	171–357	259 ± 55
Stichosome	5719–8581	6895 ± 749	5689–10 070	7990 ± 1245
Entire oesophagus	5868–9249	7200 ± 771	5914–10 241	8249 ± 1260
Length of posterior part	9722–23 172	15 168 ± 3961	5329–14 919	11 252 ± 2762
Ratio posterior: anterior ends	1.4–3.0	2.1 ± 0.5	0.8–1.6	1.33 ± 0.2
Tail	12–25	20 ± 4	13–23	18 ± 3
Vulva from oesophagus	46–107	68 ± 19	31–93	62 ± 18
Egg length	60–71	66 ± 3	57–70	65 ± 3
Egg width	28–34	31 ± 2	25–34	29 ± 3
Number of eggs	127–545	285 ± 110	27–199	99 ± 45
Vulval lips	Not elevated		Not elevated	

The micrometre (μm) is a unit of measurement.

Male (holotype, 12 paratypes and 8 type specimens): Body length 7150–18 098; maximum width 53–86. Whole oesophagus, 3805–9452 long; muscular oesophagus and stichosome, 160–395 and 3570–9081, respectively. Anterior: posterior body ratio (the distance from the head to the beginning of intestine: the distance from the beginning of intestine to the tip of the tail), 1:0.30–1:2.04. Tail 12–23 long. Spicule 749–1815 long (5–16% of body length) and slender (Figs 1F–H, 1K); proximal end of spicule frame-like form (Fig. 1I). Spicular sheath non-spinous (Figs 1J, 2G). Posterior end of body broad and with 2 membranous lateral rays (Figs 1F, 2F). Two post-cloacal papillae present (Fig. 1F).

Gravid female (allotype, 12 paratypes and 8 type specimens): body length 11 816–25 160; maximum width 54–112. Whole oesophagus 5914–10 241 long; muscular oesophagus and stichosome 171–357 and 5689–10 070, respectively. Anterior: posterior body ratio, 1:0.82–1:1.64. Tail, 13–23 long (Fig. 1B). Vulva with no elevated lips (Figs 1E) situated at 31–93 from oesophagus end; vagina directed posteriorly from vulva and uterus containing 27–199 eggs. Eggs elongate with slightly protruding polar plugs (Figs 1C, 1E) and uncleaved content, 57–70 long and 25–34 wide; egg wall thick with 2 layers: an inner hyaline layer and a thicker outer layer with fine longitudinal sculpture (Fig. 1C).

### Type materials

Holotype: mature male was deposited at the Mahidol University Museum of Natural History (Voucher no.: MUMNH-NEM0001; specimen code: SN62PMI05) was collected by Vachirapong Charoennitiwat and his team, on July 18, 2023, in the small intestine of a monocled cobra, *Naja kaouthia* (IDs: SN062 for this project; AAS089 [EL-Nk-025] for the Applied Animal Science laboratory's catalogue), at the Department of Helminthology, Faculty of Tropical Medicine, Mahidol University.

Description of holotype: body length 13 069; maximum width 66. Whole oesophagus, 7318 long; muscular oesophagus and stichosome, 305 and 7013, respectively. Stichosome with a single row of 40 stichocytes. Anterior: posterior body ratio, 1: 0.79. Tail 14 long. Spicule 1402 long (9.32% of body length) and slender; proximal end of spicule frame-like shape. Spicular sheath non-spinous. Posterior end of body broad and with two membranous lateral rays. Two post-cloacal papillae present.

Allotype: gravid female was deposited at the Mahidol University Museum of Natural History (Voucher no.: MUMNH-NEM0002; specimen code: SN62PFI07) was collected by Vachirapong Charoennitiwat and his team, on July 18, 2023, in the small intestine of a monocled cobra, *Naja kaouthia* (IDs: SN062 for this project; AAS089 [EL-Nk-025] for the Applied Animal Science laboratory's catalogue), at the Department of Helminthology, Faculty of Tropical Medicine, Mahidol University.

Description of allotype: body length 19 720; maximum width 102. Whole oesophagus 7986 long; muscular oesophagus and stichosome 327 and 7630, respectively. Stichosome with a single row of 41 stichocytes. Anterior: posterior body ratio, 1:1.47. Tail 23 long. Vulva with no elevated lips situated at 71 from oesophagus end; vagina directed posteriorly from vulva and uterus containing 135 eggs. Eggs elongate with slightly protruding polar plugs and uncleaved content, 60 long and 30 wide; egg wall thick with 2 layers: an inner hyaline layer and a thicker outer layer with fine longitudinal sculpture.

Paratypes (1–24): a total of 12 males were deposited at the Mahidol University Museum of Natural History (Voucher no.: MUMNH-NEM0003‒NEM0014; specimen code: SN18PMI01‒PMI03; SN59PMI01‒PMI03; SN62PMI01, PMI03, PMI04, PMI06, PMI07 and PMI09, respectively). A total of 12 gravid females, at the Mahidol University Museum of Natural History (Voucher no.: MUMNH-NEM0015‒0026; specimen code: S18PFI01‒PFI03; SN59PFI01‒PFI03; SN62PFI02, PFI03, PFI05, PFI06, PFI08 and PFI09, respectively).

These paratype specimens were relatively collected from the small intestine of monocled cobras (Snake IDs: SN018, SN059 and SN062 for this project; or AAS022 [EL-Nk-016], AAS089 [EL-Nk-025] and AAS069 [EL-Nk-022] for the Applied Animal Science laboratory's catalogue) by Vachirapong Charoennitiwat and his team, on September 30, 2021; November 10, 2022; and July 18, 2023, respectively. All cobra hosts were obtained in Bangkok, Thailand, but the precise collecting coordinates were not recorded. The general description includes comprehensive morphological descriptions of these paratypes.

### Diagnosis

As specified (see Table S1), *Paracapillaria* (*Ophidiocapillaria*) *siamensis* sp. nov. can be distinguished from other *Paracapillaria* species by diagnostic characteristics: body length 7150‒18 098 in males and 11 816‒25 160 in females. Maximum width 53‒86 in males and 54‒112 in females. Stichosome with a single row of 39‒45 stichocytes. Specifically, this nematode species was found in the snake intestine, frequently in the small intestine. Anterior: posterior body ratio in males, usually 1:<1 with average 1:0.9. The number of eggs in uterus is usually <199 with average 99. The eggs arrange in 1‒2 rows in uterus. Proximal end of spicule of male frame-like shape (Fig. 1I); spicule length 749‒1815. The cephalic region displayed elevated lips without protrusion (Figs 2A‒B). The body's midsection featured transverse striations on the ventral side (Fig. 2C). A dorsal stripe of bacillary band extending from the nearly beginning of the body to the cloaca (Figs 2D‒E). This bacillary band consists of irregular pattern of bacillary cells (Fig. 2D). *P. siamensis* sp. nov. primarily infected the small intestine of the snake host.

### Specific diagnosis

Among these *Ophidiocapillaria*, the newly described Thai species, *Paracapillaria siamensis* sp. nov. can be distinctly differentiated from Italian *Paracapillaria mingazzinii* (Rizzo, 1902), American *Paracapillaria colubra* (Pence, 1970), *Paracapillaria heterodontis* (Harwood, 1932), Bulgarian *Paracapillaria viperae* Biserkov *et al*., 1985, Mexican *Paracapillaria xochimilcensis* (Caballero & Cercero, 1943), Brazilian *Paracapillaria murinae* (Travassos, 1914) and Portuguese *Paracapillaria cesarpintoi* (Freitas & Lent, 1934) due to differences in vulva lip characteristics. *P. siamensis* sp. nov. is characterized by the absence of elevated vulva lips, distinguishing it from the mentioned species. Furthermore, *P. siamensis* sp. nov. differs from these species in several measured characteristics, including longer spicule length (in *P. colubra*, *P. heterodontis*, and *P. viperae*), shorter spicule length (in *P. cesarpintoi*), a larger distance between the vulva opening and the end of the oesophagus (in *P. colubra* and *P. heterodontis*), and a shorter distance between the vulva opening and the end of the oesophagus (in *P. cesarpintoi*). Importantly, *P. siamensis* sp. nov. was recorded in Thailand, Asia, while all the other species were discovered on different continents.

In addition, *P. colubra*, *P. heterodontis* and *P. murinae* were found in the oviduct, rectum and stomach of snakes, unlike *P. siamensis* sp. nov., which specifically inhabits the small intestine. Based on Moravec (1986), *P. siamensis* sp. nov. does not exhibit the same levels of characteristic variation as *Paracapillaria sonsinoi* (Parona, 1897), which includes body length of both genders, spicule length, the ratio of the anterior and posterior ends, egg length and importantly, its recorded locality in Europe and America.

Among Oriental *Ophidiocapillaria*, Taiwanese *Paracapillaria kuntzi* (Moravec and Gibson, 1986) displays smaller maximum width in both males and females (♂ 53‒86 *vs* 95‒150 and ♀ 54‒112 *vs* 122‒204) and a shorter distance between the nerve-ring and the tip of the head (♂ 23‒69 *vs* 75‒105 and ♀ 40‒69 *vs* 78‒96), compared to *P. siamensis* sp. nov. In contrast, Indian *Paracapillaria longispicula* (Sonsino, 1889) shows significantly larger body length (♂ 40 000 *vs* 7150‒18 098 and ♀ 50 000‒60 000 *vs 11 816*‒25 160), longer spicule length: (2000‒3820 *vs* 749‒1815), and a greater distance between the vulva opening and the end of the oesophagus (140 *vs* 31‒93), compared to *P. siamensis* sp. nov.

The Chinese *Paracapillaria ptyasi* (Wang, 1982) has different key characteristic compared to *P. siamensis* sp. nov: anterior spicule shape (round *vs* frame-like), the number of stichocytes (32‒34 *vs* 39‒45), the position of nerve-ring from the anterior end (40‒69 *vs* 80‒90), a shorter range of female body length (10 200‒11 900 *vs 11 816*‒25 160) and anterior part length (5000‒5200 *vs* 5914‒10 241). Within the same locality (centre Thailand) and snake host species (the monocled cobra, *Naja kaouthia*) – co-infection, *Paracapillaria najae* De, 1998, differs from *P. siamensis* sp. nov. in several aspects: anterior spicule shape (spatula-like *vs* frame-like), infected organ (oesophagus *vs* small intestine), the ratio of posterior and anterior ends in both males and females (♂ 1.2–2.5 ave. 1.8 *vs* 0.3–2.0 ave. 0.9 and ♀ 1.4–3.0 ave. 2.1 *vs* 0.8–1.6 ave. 1.33), spicule length (1437–2715 ave. 2032 *vs* 749–1815 ave. 1381), a bacillary band covered dorsolaterally *vs* dorsally and other minor characters, with the measurements of *P. najae* typically being larger than those of *P. siamensis* sp. nov. (see Table 1).

### Variation

The number of *P*. *siamensis* sp. nov. individuals, ranging from ~16 to 82 worms, displays significant morphological variation among the cobra specimens (see Table S2). Key characters that vary among the cobras include relative-to-body size and oesophagus characteristics of both male and female worms, encompassing body length and width, oesophagus lengths and other distance characters. Notably, cobra ID: SN013 exhibited relatively small parasites (average 7946.35, *n* = 4), whereas other cobra samples displayed nearly double the size in body length (14 171.67–15 788.92, *n* = 3–7). The spicule length stands out as another example of character with high variation; the worms in cobra ID: SN059 had an average spicule length of 2480.26 (*n* = 3), surpassing those in cobra IDs: SN018 and SN013, which measured 1265.00 (*n* = 3) and 969.60 (*n* = 4), respectively. This trend is also observable in other morphological characteristics. Despite the pronounced morphological diversity among *P. siamensis* sp. nov. individuals, specimens subjected to genetic analysis revealed minimal sequence differences (Fig. 2).

Sexual dimorphism, reflected in traits such as larger size in females (average 19 630.29 *vs 13 672*.54 in males), distinct vulva lips in females (Fig. 1E) and specific features of the spicular sheath in males (Figs 1G–H and J), is observed in this novel species (Table S2).

### Genetic characterization and phylogenetic position

**Figure 3.**
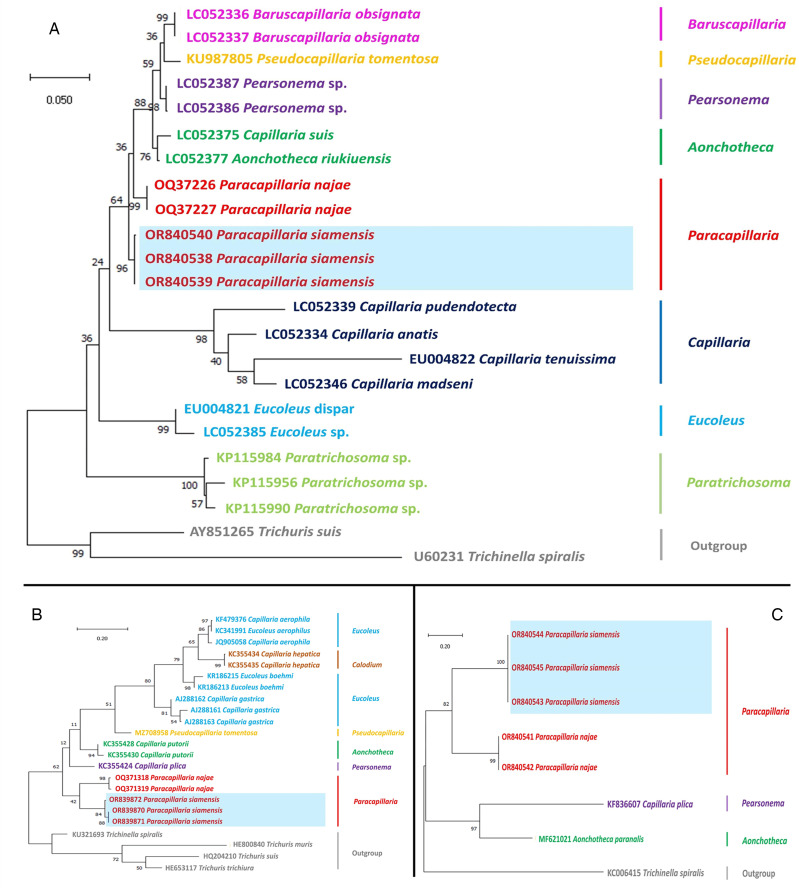
Phylogenetic analysis of capillariids based on different genetic markers: (A) *COI* gene, (B) 18S rRNA and (C) ITS1. The analyses were conducted using MEGAX with the maximum likelihood method. Branch length scale bars indicate the number of substitutions per site. Coloured lines/fonts represent genetic data from various capillariid genera sourced from GenBank, with the red line/font specifically highlighting the genus *Paracapillaria*. The blue box indicates the specimens of *Paracapillaria siamensis* sp. nov. used in the present study. Synonym remarks include *Capillaria hepatica* = *Calodium hepaticum*, *Capillaria gastrica* = *Eucoleus gastricus*, *Capillaria putorii* = *Aonchotheca putorii*, *Capillaria plica* = *Pearsonema plica*, and *Capillaria suis* = *Aonchotheca suis*.

The molecular characterization of the newly identified species, *P. siamensis* sp. nov., involved the amplification and sequencing of the nuclear 18S rDNA and ITS1 genes, as well as the mitochondrial *COI* gene. The outcomes of the phylogenetic analysis clearly indicated that *P. siamensis* sp. nov. is a distinct species within the taxonomic classification of Capillariidae. The species available for comparison included *Baruscapillaria* sp., *Pseudacapillaria* sp., *Pearsonema* sp., *Aonchotheca* sp., *Capillaria* spp., *Eucoleus* spp., *Paratrichosoma* sp. and other *Paracapillaria* sp. Specifically, the *COI* strongly suggested that *P. siamensis* sp. nov. forms a sister clade with *P. najae* (Fig. 3A), even though the 2 species infect the same snake host species. This illustrates the co-infection between both paracapillariid species. Both nuclear 18S rRNA and ITS1 also supported the differentiation of *P. siamensis* sp. Nov. from *P. najae* and other capillariid sequences available in GenBank, similar to the mtDNA result (Figs 3B–C). The genetic variation between *P. siamensis* sp. Nov. and other capillariids ranged from 14 to 25% for the *COI*, 2–14% for the 18S and 41–63% for the ITS1. The closest genetic distances were observed between *P. siamensis* sp. nov. and members of *P. najae*, with 14% for the *COI*, 2% for the 18S rRNA and 41% for the ITS1.

### Natural history

*Paracapillaria siamensis* sp. nov., appears to be a prevalent parasite in the monocled cobra, given its discovery in all 5 snake samples examined. This species, exclusively found in the small intestine of the cobras, has been documented to co-occur with *P. najae*, which predominantly resides in the oesophagus. This co-occurrence was observed consistently across all snake samples examined in the study. As a common behaviour, the worms tend to aggregate through ball-coiling within the intestinal mucus, extending throughout the entire intestinal tract and occasionally reaching into the proximal large intestine area. Importantly, visual inspection revealed no discernible lesions on the target organ.

Although the precise geographic coordinates of the sample collection sites were not recorded, the cobras were captured in suburban areas surrounding Bangkok, directly linked to their prey choices. Within the gut contents, a conspicuous presence of small conical structures resembling the teeth of lizards or rodent claws was observed under the stereomicroscope. This observation raises the possibility of intermediate hosts for the new paracapillariid species within the ecosystem.

## Discussion

*Paracapillaria siamensis* sp. nov., as per Moravec and Justine (2020), belongs to the subgenus *Ophidiocapillaria*, as it resides in the snake species, the monocled cobra, *Naja kaouthia*, found in Thailand. The key diagnostic morphology of this new species aligns with that of other *Paracapillaria* spp., including the presence of a bursa supported by lateral lobes and a non-spiny spicular sheath at the male caudal end (e.g. Moravec, 1986; Moravec *et al*., 1995; Moravec and Justine, 2020). Furthermore, the phylogenetic results from both mitochondrial and nuclear genes in this study strongly support the morphological findings, indicating that this new paracapillariid species falls within the capillariid lineage.

The newly discovered *Ophidiocapillaria* species exhibits distinct morphological differences from other species within the same subgenus, characterized by variations in morphological measurements and proportions. Key features used for classifying this new paracapillariid species include body length, body width, oesophagus length, distance from the nerve ring to the anterior end, stichosome and spicule length, among others (e.g. Moravec, 1986; Moravec and Gibson, 1986; Moravec *et al*., 1995; De, 1998; Moravec and Justine, 2020; Sakaguchi *et al*., 2020; Charoennitiwat *et al*., 2023). Several characteristics, such as the target organ of infection, type of vulva lips, shape of the proximal spicule and locality, can serve as alternatives for species identification (e.g. Moravec, 2001; Timi *et al*., 2007; Moravec and Justine, 2010 and, 2020; Melnychuk *et al*., 2020; Sakaguchi *et al*., 2020; Carvalho *et al*., 2023). Despite the distinctiveness of many species based on those characteristics, a few original papers on *Paracapillaria* lacked morphological details, resulting in difficulties in the identification of species, particularly *Paracapillaria ptyasi* found in the southern part of China. This species exhibited minimal morphological differences from *P. siamensis* sp. nov., except for a few measurements i.e. nerve-ring position, stichocytes numbers, anterior part length, female body length and importantly, the spicule shape, a key factor in *Paracapillaria*'s identification (*sensu* Moravec and Justine, 2020; Carvalho *et al*., 2023). The spicule shape was markedly different between these 2 species – round for *P. ptyasi* and frame-like for *P. siamensis* sp. nov.

Additional evidence for the differentiation of these 2 worm species lies in the isolation of their hosts and the host distribution (the monocled cobra, *Naja kaouthia*, *vs* the Oriental rat snake, *Ptyas mucosa*). Moreover, there are reports of geographical distribution separation in the rat snake (genus *Ptyas*) between China and Thailand (Gernot and Sjon, 2013). This separation is attributed to the high elevation of the Himalayan Mountain ranges, suggesting that parasites restricted to their hosts might not cross the border. Similar isolation of reptiles is observed in other Colubrid species (Bain and Hurley, 2011), such as the keelback snake, *Rhabdophis subminiatus* (Liu *et al*., 2021) and *Dendrelaphis* sp. (Biakzuala *et al*., 2022).

However, species infected within the same snake host, like *P. siamensis* sp. nov. and *P. najae* are distinguished by the described morphological measurements and ratios in Table 1, as well as descriptive characteristics such as proximal spicule shape (frame-like for *P. siamensis* sp. nov. *vs* spatula-like for *P. najae*, *sensu* Charoennitiwat *et al*., 2023) and the organ of infection (the intestine for *P. siamensis* sp. nov. *vs* the oesophagus for *P. najae*). Nevertheless, these characteristics, which closely resemble each other between the 2 species, pose challenges in species identification when the parasites are isolated from the host body and in low numbers. Therefore, microscopic structure and molecular genetic studies play a crucial role in species identification for paracapillariid worms, given their high morphological variation (Charoennitiwat *et al*., 2023), potentially leading to species misidentification. The microscopic surface images clearly display variation in the position of the bacillary band between the 2 species. The genetic information derived from the mt *COI*, as well as the nuclear 18S rDNA and ITS1 (slowly evolving nuclear genes, Mallatt *et al*., 2004) markers, provides valuable insights into the identification, confirming the genetic distinctiveness of *P. siamensis* sp. nov. from *P. najae*. This emphasizes the importance of genetic data for the species identification of *Paracapillaria*, although the available data in GenBank on *Paracapillaria* species is still limited.

Despite the considerable morphological variation observed in *P. siamensis* sp. nov., the genetic sequence exhibited minimal differences. This suggests that the observed morphological differentiation may arise from other factors, such as environmental influences (e.g. Thompson and Lymbery, 1990; Benesh and Kalbe, 2016). Therefore, considering alternative sources of information, in addition to morphological characteristics, for species identification may be advisable.

The co-infection involving 2 paracapillariid species: *P. siamensis* sp. nov. and *P. najae*, within the same snake host, the monocled cobra, is documented for the first time in this study. However, such occurrences are not unique, as evidenced in other parasitic helminth groups (Behnke *et al*., 2001), such as *Angiostrongylus* (Watthanakulpanich *et al*., 2021), *Trichinella* (Marucci *et al*., 2022), *Taenia* (Anantaphruti *et al*., 2007; Devleesschauwer *et al*., 2012; Shin *et al*., 2019) and *Trichuris* (Areekul *et al*., 2010). *P. siamensis* sp. nov. and *P. najae* exhibit parasitism in different organs of the same host, potentially mitigating competition between the 2 species (Stancampiano *et al*., 2010; Venter *et al*., 2022; Presswell *et al*., 2023). This phenomenon departs from the norm observed in other parasitic helminth groups, where co-infections occur regularly.

*P. siamensis* sp. nov., appearing in the adult stage, infects the monocled cobra, a generalist species predominantly found in the central lowland region of Thailand (Cox *et al*., 2012; Ratnarathorn *et al*., 2019 and, 2023). It is plausible that transmission of the parasite species occurs through various prey items consumed by the cobra, such as rodents, chicks, amphibians or other reptiles (Cox *et al*., 2012; Kalki *et al*., 2022). Given the capability of paracapillariid species to infect multiple hosts (Charoennitiwat *et al*., 2023), a further investigation involving their prey and other generalist snake species, such as rat snakes (*Ptyas* spp.) and the Siamese spitting cobra (*Naja siamensis*), which share similar prey preferences with the monocled cobra (Cox *et al*., 2012), may contribute to a more comprehensive understanding of the natural history aspects, including the life cycle of this newly described species.

In conclusion, both morphological and molecular characterizations provide robust evidence supporting the identification of a new species within the subgenus *Ophidiocapillaria*. This discovery prompts formally naming of the species *Paracapillaria* (*Ophidiocapillaria*) *siamensis* sp. nov. The distinct characteristics observed in this species set it apart from closely related species within the *Ophidiocapillaria* subgenus. Additionally, the study unveils a noteworthy aspect of co-infection involving *P. siamensis* sp. nov. and *P. najae* in the monocled cobra, *Naja kaouthia*, collected from the same locality. This marks the first documented evidence of potential host sharing within the *Paracapillaria* group.

## Supporting information

Charoennitiwat et al. supplementary material 1 Charoennitiwat et al. supplementary material

Charoennitiwat et al. supplementary material 2 Charoennitiwat et al. supplementary material

## Data Availability

The data that support the findings of this study are available from the first and corresponding authors upon reasonable request.

## Supplementary material

The supplementary material for this article can be found at https://doi.org/10.1017/S0031182024000404.
